# Intrasinusoidal Hodgkin Lymphoma; A Mimic of Anaplastic Large Cell Lymphoma

**DOI:** 10.1155/2021/6737829

**Published:** 2021-10-26

**Authors:** John R. Krause, James Fowler, Javed I. Gill

**Affiliations:** ^1^Department of Pathology, Section of Hematopathology Baylor University Medical Center at Dallas, Dallas, TX 75246, USA; ^2^Central Texas ENT Associates, Brownsville, TX 76801, USA

## Abstract

The distinction between classical Hodgkin lymphoma (HL) and anaplastic large cell lymphoma (ALCL) is not problematic in most instances. In rare situations, HL may present with a sinusoidal infiltrative pattern that may mimic ALCL. It is important to use a battery of immunohistochemical stains to differentiate between these two entities as therapy and clinical behavior are different. We present a case of a young woman who presents with the very unusual intrasinusoidal infiltrative pattern.

## 1. Introduction

The distinction between Hodgkin lymphoma (HL) and anaplastic large cell lymphoma (ALCL) is not problematic in most instances. However, in rare situations, HL may present with a sinusoidal infiltrative process that may mimic ALCL. It is important to differentiate between these two entities as therapy and clinical behavior are different. We present a case of a young woman who presents with HL exhibiting the very unusual intrasinusoidal infiltrative pattern.

## 2. Case Summary

**Figure 1:**
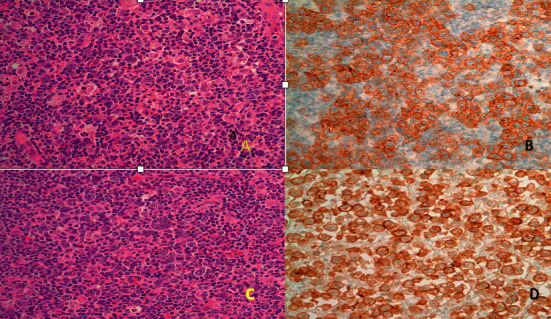
(a) HL intrasinusoidal infiltrate. H&E x400. (b) HL. CD30 stain x400. (c) ALCL intrasinusoidal infiltrate. H&E x400. (d) ALCL. CD30 stain x400. HL, Hodgkin lymphoma. ALCL, anaplastic large cell lymphoma.

A 37-year-old woman presented with marked tonsillar enlargement and right neck swelling of about 3 months duration. Biopsies were performed which revealed a large atypical cell neoplasm infiltrating the right cervical node and right tonsil in an intrasinusoidal pattern (Figure 1(a)). The initial morphologic impression was an anaplastic large cell lymphoma. The CD30 stain was strongly positive (Figure 1(b)). However, the cells were negative for CD45, T cell markers, including CD43, ALK 1, and CD15. The atypical cells did stain positive for MUM and weakly positive for PAX5. A few of the atypical cells were positive for EBER. These immunohistochemical findings are consistent with classic HL. Further Imaging studies revealed only the right tonsil and right cervical lymph nodes to be involved (Stage IIA). Pertinent laboratory findings included a hemoglobin of 11.7 g/dl, hematocrit of 35%, and platelet count of 267 × 10^3^/ul. The WBC count was 9 × 10^3^/ul with a differential of 54% neutrophils, 4 bands, 32 lymphocytes, 6 monocytes, and 4 eosinophils. The glucose was mildly elevated at 115 mg/dl and the LDH was 440 U/L (normal 85–245). Other laboratory parameters were within normal limits. A bone marrow biopsy was negative with no evidence of lymphoma.

## 3. Discussion

In the vast majority of cases, the distinction between HL and ALCL is not a problem [1]. In rare instances such as this case, the morphology and sinusoidal pattern of infiltration favor an ALCL. A striking intrasinusoidal infiltrating pattern is a very unusual finding in HL and few cases have been reported [2, 3]. In the earlier European literature [4], the intrasinusoidal spread of tumor cells was regarded as a very important criteria in favor of what was then called Hodgkin's like anaplastic large cell lymphoma. It is widely accepted that HL and ALCL have many similarities and this variant may represent an area of overlap [2, 5]. A case of ALCL is illustrated in Figures 1(c) and 1(d) to show the morphologic similarities and CD30 staining pattern to the intrasinusoidal variant of Hodgkin lymphoma. It is very important to do a battery of immunohistochemical stains to help distinguish between the two. Both entities are CD30 positive and while HL will express CD15 in about 75% of the cases. ALCL may also express CD15 in 0–25% of cases, so these two classic markers for Hodgkin lymphoma may not differentiate the two entities. A battery of immunohistochemical stains which are shown in Table 1 will help to distinguish the two as well as the presence of a T-cell receptor rearrangement and *t*(2; 5) cytogenetic presence that are found in ALCL and not in HL. From the above and Table 1, it may be seen that there are some overlaps in these diseases and if too much reliance is placed on the histological features and the presence of only CD30 and CD15 stains, a mistaken diagnosis may result [6]. The use of the immunohistochemistry stain PAX5 is useful as the Reed Sternberg cells of HL are weakly positive and ALCL cells are negative. Likewise, the IRF/MUM stain is positive in HL and negative in ALCL. It is important to point out that while these lesions have some overlaps, if sufficient stains are not performed and the appearance may not trouble an experienced hematopathologist, it is a potential area of confusion for the general pathologist who may not see many lymph node cases and/or who may have a limited number of immunohistochemical stains and genetic tests available. In this situation, the assistance of an experienced hematopathologist should be considered.

With complete immunophenotypic and genetic studies that are now currently available, anaplastic large cell lymphoma can be distinguished from classic Hodgkin lymphoma in virtually all cases. This distinction is important as these two diseases have different clinical behaviors, prognosis, and treatment options.

## Figures and Tables

**Table 1:** Immunophenotypic and genetic studies.

Anaplastic large cell lymphoma	Classic Hodgkin lymphoma
Immunophenotype	
T/null	T-cell (rare 5%)
CD15−/+ (0–20%)	CD15+ (75%)
PAX5 −	PAX5 weakly + (90%)
CD45 usually +	CD45 usually −
EMA + (60–100%)	EMA – (rarely + 0–5%)
CD43 +	CD43 usually −
IFR/MUM −	IFR/MUM +
ALK 1 may be + in some cases	ALK 1 −
EBER + (5–20%)	EBER + (15–75%)

Genetic studies	
T-cell receptor gene rearranged	
*t*(2,5)-NPM/ALK rearrangement	
